# Neutralization Serotyping of BK Polyomavirus Infection in Kidney Transplant Recipients

**DOI:** 10.1371/journal.ppat.1002650

**Published:** 2012-04-12

**Authors:** Diana V. Pastrana, Daniel C. Brennan, Nicolas Çuburu, Gregory A. Storch, Raphael P. Viscidi, Parmjeet S. Randhawa, Christopher B. Buck

**Affiliations:** 1 Laboratory of Cellular Oncology, National Cancer Institute, Bethesda, Maryland, United States of America; 2 Washington University School of Medicine, St. Louis, Missouri, United States of America; 3 Department of Pediatrics, Johns Hopkins Medical Center, Baltimore, Maryland, United States of America; 4 Department of Pathology, University of Pittsburgh Medical Center, Pittsburgh, Pennsylvania, United States of America; University of Michigan, United States of America

## Abstract

BK polyomavirus (BKV or BKPyV) associated nephropathy affects up to 10% of kidney transplant recipients (KTRs). BKV isolates are categorized into four genotypes. It is currently unclear whether the four genotypes are also serotypes. To address this issue, we developed high-throughput serological assays based on antibody-mediated neutralization of BKV genotype I and IV reporter vectors (pseudoviruses). Neutralization-based testing of sera from mice immunized with BKV-I or BKV-IV virus-like particles (VLPs) or sera from naturally infected human subjects revealed that BKV-I specific serum antibodies are poorly neutralizing against BKV-IV and vice versa. The fact that BKV-I and BKV-IV are distinct serotypes was less evident in traditional VLP-based ELISAs. BKV-I and BKV-IV neutralization assays were used to examine BKV type-specific neutralizing antibody responses in KTRs at various time points after transplantation. At study entry, sera from 5% and 49% of KTRs showed no detectable neutralizing activity for BKV-I or BKV-IV neutralization, respectively. By one year after transplantation, all KTRs were neutralization seropositive for BKV-I, and 43% of the initially BKV-IV seronegative subjects showed evidence of acute seroconversion for BKV-IV neutralization. The results suggest a model in which BKV-IV-specific seroconversion reflects a *de novo* BKV-IV infection in KTRs who initially lack protective antibody responses capable of neutralizing genotype IV BKVs. If this model is correct, it suggests that pre-vaccinating prospective KTRs with a multivalent VLP-based vaccine against all BKV serotypes, or administration of BKV-neutralizing antibodies, might offer protection against graft loss or dysfunction due to BKV associated nephropathy.

## Author Summary

Serological studies have shown that nearly all humans are chronically infected with BK polyomavirus (BKV). The infection isn't usually associated with noticeable symptoms. However, opportunistic replication of BKV in therapeutically immunosuppressed kidney transplant recipients (KTRs) can lead to dysfunction or loss of the engrafted kidney. BKV associated nephropathy can occur even in KTRs with high levels of anti-BKV antibodies that might be expected to neutralize the virus. In this report we provide a possible explanation: we show there are at least two BKV genotypes, which are distinct serotypes with respect to antibody-mediated neutralization. Using a novel neutralization-based approach, we found that about half of 108 KTRs did not have detectable levels of antibodies capable of neutralizing BKV genotype IV (BKV-IV) at the time of transplantation. Of these initially BKV-IV naïve KTRs, about half experienced acute BKV-IV specific seroconversion during the first year after transplantation. This likely reflects a de novo BKV-IV infection arising from the engrafted kidney. In a pilot study, we show that recombinant BKV-IV VLPs can induce high levels of BKV-IV-neutralizing antibodies in vaccinated animals. Our results suggest that administration of a BKV VLP-based vaccine to prospective KTRs might protect against the development of opportunistic BKV replication.

## Introduction

The process of kidney transplantation has been revolutionized since the first successful case in identical twins more than 5 decades ago [1], [2]. Since then, the use of immunosuppressants such as cyclosporine has made renal allografts a viable clinical option [3], but the process still has many challenges, including the management of chronic and acute immune-mediated rejection of the allograft, nephrotoxicity from immunosuppressants and antiviral drugs, and controlling opportunistic infections. To balance these factors, clinical guidelines for the treatment of kidney transplant recipients (KTRs) generally suggest the use of intensive immunosuppression during the initial stages of the process, followed by a diminished dose of immunosuppressants if there are no signs of acute rejection by 2–4 months after transplantation [4].

In addition to the potential problem of immunological rejection of the allograft, between 1 and 10% of KTRs develop nephropathy associated with a non-enveloped DNA virus species called BK polyomavirus (BKV or BKPyV) [5]–[8]. Serological and PCR-based studies indicate that nearly all human beings are chronically infected with BKV [9], [10]. Although chronic BKV infection of the urinary tract is not known to be associated with overt clinical symptoms in healthy individuals, opportunistic replication of the virus in KTRs can lead to graft dysfunction or loss [11]. BKV can also cause a bladder condition known as hemorrhagic cystitis in bone marrow transplant recipients and in cancer patients treated with the immunosuppressant cyclophosphamide [12], [13]. Clinical guidelines for these conditions therefore recommend regular monitoring of serum or urinary BKV viral load and reduction of immunosuppression if signs of uncontrolled BKV replication are observed [4].

In pediatric KTRs, being BKV seronegative prior to transplantation correlates with the risk of developing BKV associated nephropathy (BKVN) [14]. In adult KTRs, at least one study suggested a significant correlation between donor BKV seroreactivity and the risk of urinary shedding of BKV in KTRs [15]. However, BKV seroprevalence is high in most adult populations and a variety of other studies have not uncovered clear correlations between KTR seroresponsiveness to BKV and resistance to BKV viremia or BKVN [16]–[18] and reviewed in [19]. Monitoring of pre-transplant BKV serology is not usually performed, based largely on the idea that it is not an effective indicator of susceptibility to BKVN. This logic has also been used to infer that vaccine-induced boosting of BKV-specific antibody responses in prospective KTRs would be unlikely to confer protection against BKVN.

BKV isolates can be grouped into four genetically distinct subspecies (genotypes) [20]–[22]. While chronic infection with BKV genotype I (BKV-I) is believed to be common almost to the point of ubiquity in all human populations worldwide, PCR-based studies suggest that BKV genotypes II, III, and IV only infect a minority of adults. The incidence of BKV-IV infection varies among different populations, with estimates ranging from <5% of BKV isolates detectable in urine specimens contributed by Sub-Saharan African subjects to 54% of detected isolates from Northeast Asian subjects [23]. The prevalence of BKV genotypes II and III appears to be lower than BKV-IV [9]. However, a complicating factor in these studies is that many commonly-used PCR primer pairs detect BKV genotype I more efficiently than other genotypes [24].

The extent of serological cross-reactivity among BKV genotypes is unclear. Prior serological studies of KTRs have monitored BKV-specific serum antibody responses using ELISAs where the target antigens are recombinant virus-like particles (VLPs) assembled from the BKV major capsid protein VP1. A recent study [25] using VLPs based on a BKV-III isolate reported serological findings similar to earlier studies using BKV-I VLPs [19]. Furthermore, one VLP-based study found a significant correlation between BKV-II and BKV-III seroresponsiveness in human subjects [26].

Although past serological studies might seem to suggest a lack of distinct BKV serotypes, a limitation of VLP ELISAs is that they simultaneously detect antibodies that can neutralize BKV infectivity and non-neutralizing antibodies. Viruses are thought to be under greater selective pressure to accumulate evasive mutations in capsid epitopes recognized by antibodies that can neutralize infectivity. In contrast, there is presumably less selective pressure for viruses to accumulate mutations in virion surfaces recognized by antibodies that don't neutralize infection. Consequently, epitopes bound by non-neutralizing antibodies are more likely to be conserved among related viral genotypes, while neutralizable epitopes are more likely to be divergent. This poses a problem for ELISA methodology, since measurement of non-neutralizing antibody binding may obscure the existence of a subset of serotype-specific neutralizing antibodies. Consistent with this theory, we have previously shown that serological assays that monitor antibody-mediated neutralization of a different family of non-enveloped viruses, the *Papillomaviridae*, offer a more effective way to discriminate among human papillomavirus (HPV) serotypes than VLP-based ELISAs [27]. Neutralization-based serological methods also appear to offer an accurate reflection of the clinical efficacy of recently developed VLP-based vaccines against HPV [28], [29].

In a 1989 study using various cell culture-adapted BKV isolates, Knowles and colleagues demonstrated that serum antibodies from animals immunized with virions of one BKV genotype are less effective for in vitro neutralization of BKV isolates from the other three genotypes [22]. Whether this serological difference also holds true for people with chronic BKV infections is not known. For example, some people with HIV-1 infections eventually develop antibody responses capable of blocking the infectivity of a wide range of HIV-1 genotypes. Such broadly cross-neutralizing antibody responses generally take many years to develop and are not typically observed in acutely immunized animals (reviewed in [30] and [31]).

To investigate whether BKV genotypes can be divided into distinct neutralization serotypes in naturally infected humans, we developed high-throughput serological assays to monitor antibody-mediated neutralization of the infectivity of BKV-I and BKV-IV reporter vectors (pseudovirions). Our results show that BKV-I and BKV-IV are distinct serotypes with respect to functionally neutralizing serum antibodies.

Using neutralization assay methodology, we monitored the development of BKV-I and BKV-IV seroresponsiveness in a cohort of KTRs. The data show that a substantial fraction of KTRs who lacked detectable BKV-IV neutralizing antibody responses one week after transplantation experienced acute BKV-IV-specific seroconversion during the first year after transplantation. This may reflect a *de novo* BKV-IV infection arising from the engrafted kidney. The findings raise the possibility that VLP-based vaccination of candidate organ transplant recipients who are initially naïve against specific BKV serotypes might confer protection against pathological forms of BKV replication that are sometimes associated with the implementation of immunosuppressive therapy.

## Results

### BKV type-specificity of ELISAs versus neutralization-based serological assays

**Figure 1:**
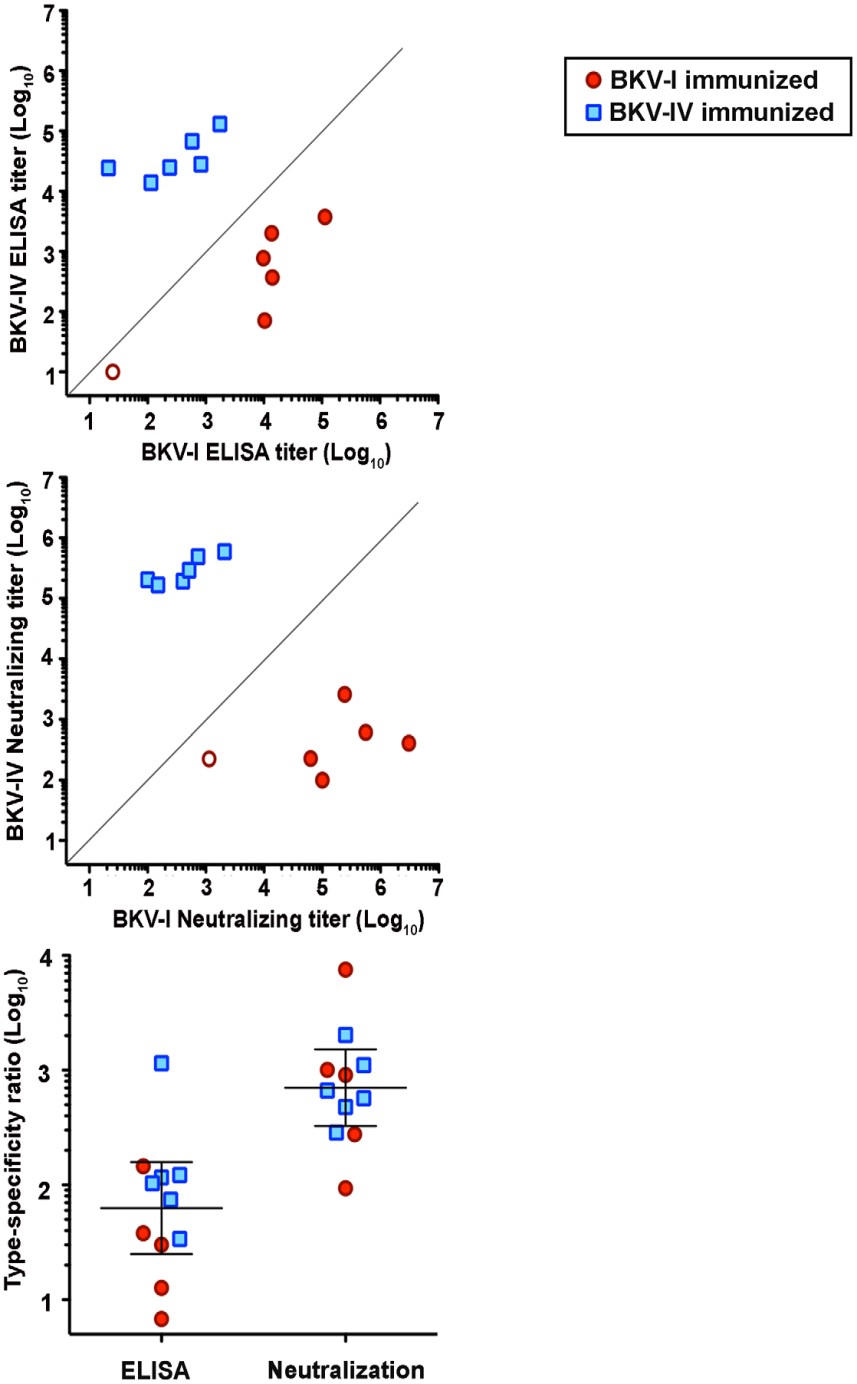
Immunization of mice with BKV-I and BKV-IV VLPs. Six mice were immunized with BKV-I (red circles) or BKV-IV (blue squares) VLPs. In the top panel, sera were serially diluted and tested in separate BKV-I (x axis) or BKV-IV (y axis) VLP ELISAs. A data point from one relatively non-responsive animal is shown as an open circle. The middle panel depicts BKV type-specific neutralizing titers for the same set of mice. The gray diagonal line depicts a theoretical 1∶1 correlation (i.e., perfect cross-reactivity) between BKV-I and BKV-IV titers. The bottom panel shows the ratio of the neutralizing titer for the BKV type administered as a vaccine versus the neutralizing titer for the heterologous BKV type for individual animals. The wide bar represents the geometric mean and the error bars show the 95% confidence interval. Since the BKV-IV ELISA titer of the non-responsive animal could not be calculated, this animal was excluded from the analysis in the bottom panel.

Virus-like particles (VLPs), including BKV VLPs [32], can be potently immunogenic when administered as vaccines in animal model systems (reviewed in [33]). To generate BKV VLP vaccine immunogens, we co-expressed the VP1, VP2 and VP3 capsid proteins of the BKV-I isolate KOM-5 or the BKV-IV isolate A-66H via transfection of the human embryonic kidney-derived cell line 293TT [34]–[37]. The resulting VLPs were purified by ultracentrifugation through Optiprep gradients. Five µg of purified BKV-I or BKV-IV VLPs were mixed with complete Freund's adjuvant and administered separately to sets of six mice. Serum antibody responses were assayed four weeks after a single vaccination using ELISA plates coated separately with BKV-I or BKV-IV VLPs. All the mice showed high titer serum antibody responses in ELISAs against the cognate BKV VLP type, except for one BKV-I vaccinated mouse which appeared to be relatively non-responsive (Figure 1, top panel). The sera exhibited varying amounts of cross-reactivity against the non-cognate BKV. The average ratio of cognate BKV to non-cognate BKV titer was 21 for mice immunized with BKV-I and 110 for mice immunized with BKV-IV (Figure 1, bottom panel).

It has recently become possible to generate reporter vectors (also known as pseudoviruses) based on BKVs [35], [36]. These recombinant production systems made it possible for us to generate infectious capsids composed of the VP1/2/3 capsid proteins of BKV primary isolates of genotypes I and IV that are not otherwise culturable. Using reporter vector-based assays, we titered the neutralizing potency of sera from BKV-I or BKV-IV vaccinated mice. As expected, the neutralization assays showed a significantly greater degree of BKV type-specificity compared to the ELISAs. The median cognate versus non-cognate neutralizing titer ratio was 910 for mice immunized with BKV-I and 620 for mice immunized with BKV-IV (Figure 1). A comparison of ELISA values to neutralization assay values is shown in Figure S1.

To test the possibility that a booster vaccination might alter the degree of cross-neutralization of the two BKV types, we administered the mice a second dose of cognate VLPs in incomplete Freund's adjuvant one month after priming. We then performed repeat serology a total of two months after the initial priming dose. Hyperimmune sera from the boosted animals showed neutralizing titer ratios similar to the initial testing (data not shown), suggesting that boosting did not have a major effect on cross-neutralization profiles.

### Anti-BKV serological titers in healthy adults

**Figure 2:**
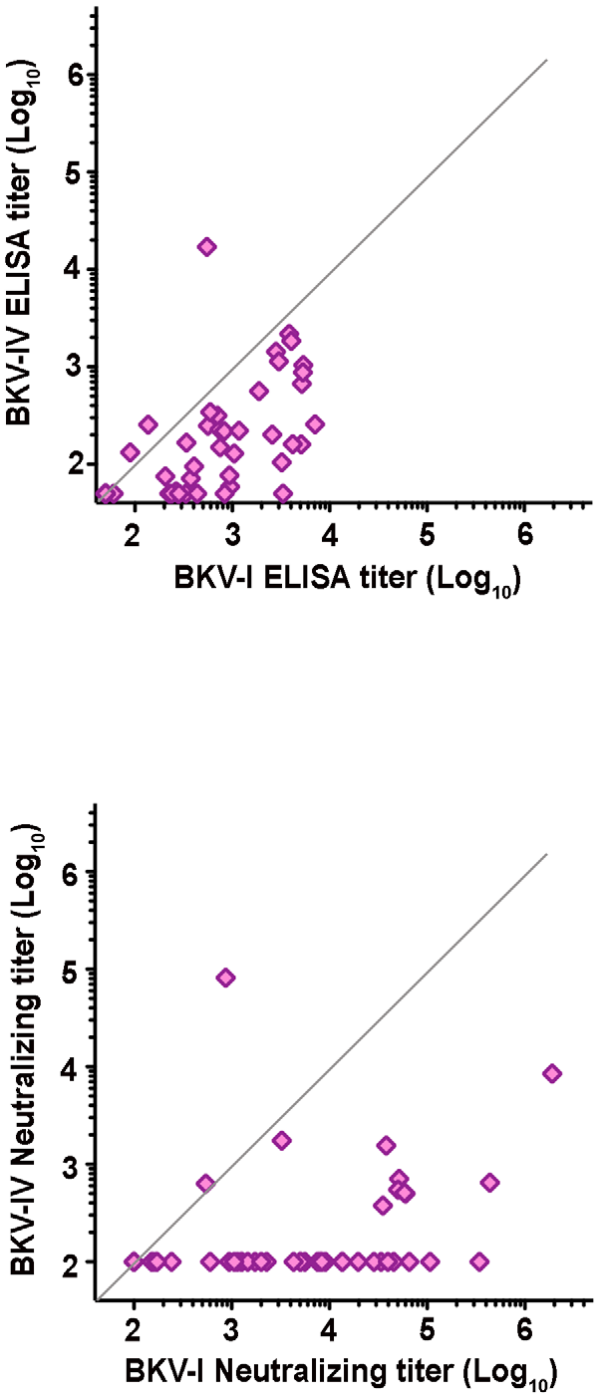
Analysis of sera from healthy adults. Sera from 48 healthy adults were evaluated for BKV type-specific serological titers. The upper panel shows BKV-I and BKV-IV titers evaluated by ELISA. The lower panel shows neutralizing titers.

Sera from 48 healthy adults with a median age of 52.5 years were assessed for reactivity to BKV-I and BKV-IV in ELISAs. Eighty-three percent of the volunteers were seropositive in the BKV-I ELISA (Figure 2, top panel), a prevalence similar to what has been reported in the literature [9], [10]. Sixty-five percent of volunteers scored seropositive in the BKV-IV ELISA. This is in contrast to the purportedly much lower prevalence of BKV-IV infection, but is consistent with the possibility that the BKV-IV ELISA detects cross-reactive antibodies elicited by BKV-I infection (and perhaps vice versa) [31]. The significant correlation between individual subjects' BKV-I and BKV-IV ELISA titers (Spearman r = 0.69, p<0.0001) is also consistent with the possibility that the BKV-I and BKV-IV ELISAs exhibit a significant degree of cross-reactivity when used for analysis of human sera.

We next applied the BKV-I and BKV-IV neutralization assays to the human serum samples. Only 3 volunteers (6%) were negative for BKV-I neutralization, while 37 (77%) volunteers scored seronegative in the BKV-IV neutralization assay (Figure 2, bottom panel). The increased rate of seropositivity for BKV-I compared to ELISA suggests that the neutralization assay is more sensitive. In contrast to the ELISA results, there was not a statistically significant correlation between the subjects' BKV-I and BKV-IV neutralizing titers. There were two individuals with BKV-I neutralizing titers of >100,000 whose sera did not detectably neutralize BKV-IV at the lowest tested dilution (1∶100). This indicates that these individuals displayed BKV type-specificity ratios of at least 1,000. Overall, the results for the human sera appear to confirm the observations using murine sera, showing that neutralization assays offer a greater degree of sensitivity and specificity for serological analysis of exposure to BKV-I and BKV-IV.

### Sequence analysis

To gain insight into which VP1 amino acids might dictate BKV neutralization serotypes, we aligned full-length non-identical VP1 peptide sequences available via GenBank (Figure S2). With respect to the BKV-I consensus, BKV-IV isolates tend to carry a variety of substitutions: E61N, N62D, F66Y, K69R, S71T, N74T, D75A, S77D, E82D, Q117K, H139N, I178V, F225Y, A284P, R340Q, K353R, and L362V. Mapping of these BKV-I/BKV-IV variant residues onto homologous positions in the X-ray crystal structures of JCV [38] and SV40 [39] suggests that, with the exception of positions 117, 225, 284, and 340, each of these BKV-I/BKV-IV variant residues is likely to be exposed on the exterior surface of the capsid. With the exception of residues 353 and 362, which are exposed along the floor of the canyons between capsomer knobs, all the exposed variations map to sites on the apical surface and apical rim of the capsomer knob. Many of the variations are adjacent to residues predicted to be involved in binding the cellular glycolipids that serve as receptors during BKV infectious entry [40], [41]. This is consistent with the idea that BKV-I/BKV-IV variations may alter epitopes recognized by antibodies that neutralize infectivity via steric occlusion of the receptor binding site.

In addition to the differences between BKV-I and BKV-IV, we noted several positions that differ stereotypically among BKV-I subtypes. For example, BKV subtype Ib-2 isolates tend to carry V42L, E82D, D175E, V210I, R340K, and L362V differences, with respect to subtypes Ia and Ib-1. Likewise, subtype Ic isolates frequently carry E20D, F225L, and R340K differences. Although these intra-genotype-I surface variations are chemically subtle, it is conceivable that the differences reflect selective pressure to escape neutralizing antibodies. An important goal of future studies will be investigation of the possibility that BKV genotype I encompasses more than one neutralization serotype. It will also be interesting to learn whether the reportedly less prevalent BKV genotypes II and III are serologically distinct from one another and/or distinct from genotypes I and IV.

### BKV type-specific seroconversion among kidney transplant recipients

**Figure 3:**
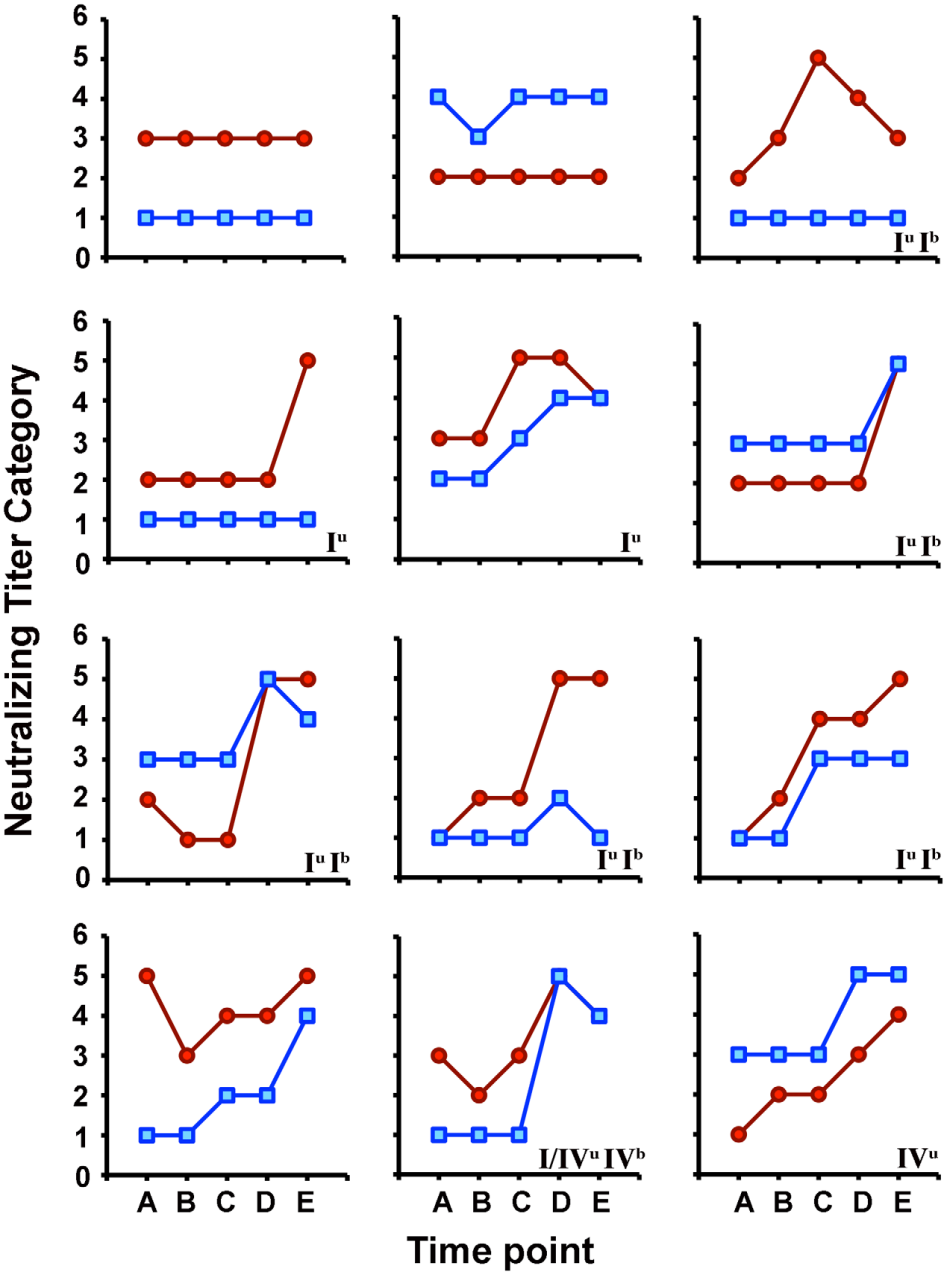
BKV-I and BKV-IV serological patterns in kidney transplant recipients. Sera from kidney transplant recipients were titered for the presence of BKV-I (red circles) or BKV-IV (blue squares) neutralizing antibodies. The neutralizing titer categories shown on the y axis are defined as 1) <95% neutralization at a serum dilution of 1∶100, 2) ≥95% neutralization at 1∶100, 3) ≥95% neutralization at 1∶500, 4) ≥95% neutralizing at 1∶5,000, and 5) ≥95% neutralizing at 1∶50,000. Sera were collected at 5 different time points (x axis) spanning roughly 1, 4, 12, 26, and 52 weeks post-transplantation, designated A-E. In each panel, the notations in the bottom right corner represent the BKV genotype (I or IV) observed in the patient's urine (superscript u) or blood (superscript b). The subject denoted I/IV^u^ showed urinary shedding of BKV-I at week 5 and urinary shedding of BKV-IV at week 16. The patterns of 12 representative patients are shown. Results for all 108 study subjects are shown in Figure S3.

Sera collected from 108 KTRs at time points of roughly 1, 4, 12, 26 and 52 weeks post-transplantation were tested using the BKV-I and BKV-IV neutralization assays. Testing of more than 500 samples at a full set of 10 serial dilutions would be expensive and logistically challenging. Therefore each serum sample was tested at 4 dilutions: 100, 500, 5,000, and 50,000. Because of this lack of full serial dilution, we elected to use a more stringent 95% neutralization cutoff of for individual data points. Neutralization assay results for individual subjects are shown in Figures 3 and S3. At study entry only 5 (5%) patients scored seronegative (i.e., <95% neutralizing at the 1∶100 serum dilution) in the BKV-I neutralization assay (Table 1). In contrast, there were 53 (49%) initially BKV-IV seronegative patients.

**Table 1:** 

Negative at entry (% of total)		Seroconversion (% of initial negatives)		Stringent Seroconversion (% of initial negatives)	
BKV-I	BKV-IV	BKV-I	BKV-IV	BKV-I	BKV-IV
5 (5%)	53 (49%)	5 (100%)	23 (43%)	5 (100%)	12 (23%)

In an initial analysis, seroconversion was defined as a change from seronegative at study entry to at least 95% neutralization at the 1∶500 serum dilution at any subsequent time point. By this standard, all 5 (100%) of the initially BKV-I seronegative patients seroconverted for BKV-I, while 23 (43%) of the initially BKV-IV seronegative patients seroconverted for BKV-IV (Table 1).

The average BKV type-specificity ratio for sera from immunized mice was 1,359 (Figure 1). Two human subjects likewise showed type-specificity ratios >1000 (Figure 2). To address the possibility that BKV-IV neutralization might be partly attributable to cross-reactivity of high titer antibody responses elicited by BKV-I, we applied a more stringent definition of seroconversion, in which the ratio of the BKV-I titer versus the BKV-IV titer must be 1,000 or less to be considered a clear type-specific seroconversion event. Using these stricter criteria, 12 (23%) of the initially BKV-IV negative patients seroconverted within a year of transplantation (Table 1).

Based on the results shown in Figures 1 and 2, the occurrence of BKV type-specificity ratios of 10 or less seems highly unlikely. Five patients (5%) underwent BKV-IV-specific seroconversion by the extremely strict criterion of having a BKV-I to BKV-IV titer ratio ≤10.

A previous study of this set of subjects used nested PCR followed by restriction fragment analysis to determine which BKV genotype was shed in the urine or blood of a subset of patients who became viruric during the course of the study [15]. Two of four patients previously found to have shed BKV-IV DNA in their urine during or after the onset of viruria seroconverted for BKV-IV neutralization by the extremely strict definition of seroconversion (Figures 3 and S3). Interpretation of the BKV genotyping data is restricted by two caveats. First, the nested PCR method used for the genotyping is likely to detect only the most abundant BKV genotype present in the sample. This problem has recently been highlighted by Luo and colleagues in a report showing that BKV-IV and chimeric quasispecies containing BKV-IV-related sequences are often present as minority sequences in urine samples from KTRs and healthy subjects with viruria [42]. An additional problem for BKV genotype analysis is that virions found in blood or urine may originate from the recipient's bladder epithelium [43]–[45] or original kidneys. Such virions might not reflect the infection status of the engrafted kidney. Interestingly, Randhawa and colleagues have reported a higher prevalence of BKV genotype IV (5/25 (20%)), as well as ambiguous BKV genotypes (5/25 (20%)) in biopsy material from engrafted kidneys affected by interstitial nephritis [46]. Establishing clear relationships (or lack thereof) between BKV type-specific serological titer and the presence of various BKV genotypes in patients at risk of BKVN will likely require deep sequencing of BKV DNA amplified using primers that target conserved portions of the BKV genome [24]. Ideally, such analyses would include biopsy specimens from engrafted kidneys.

**Figure 4:**
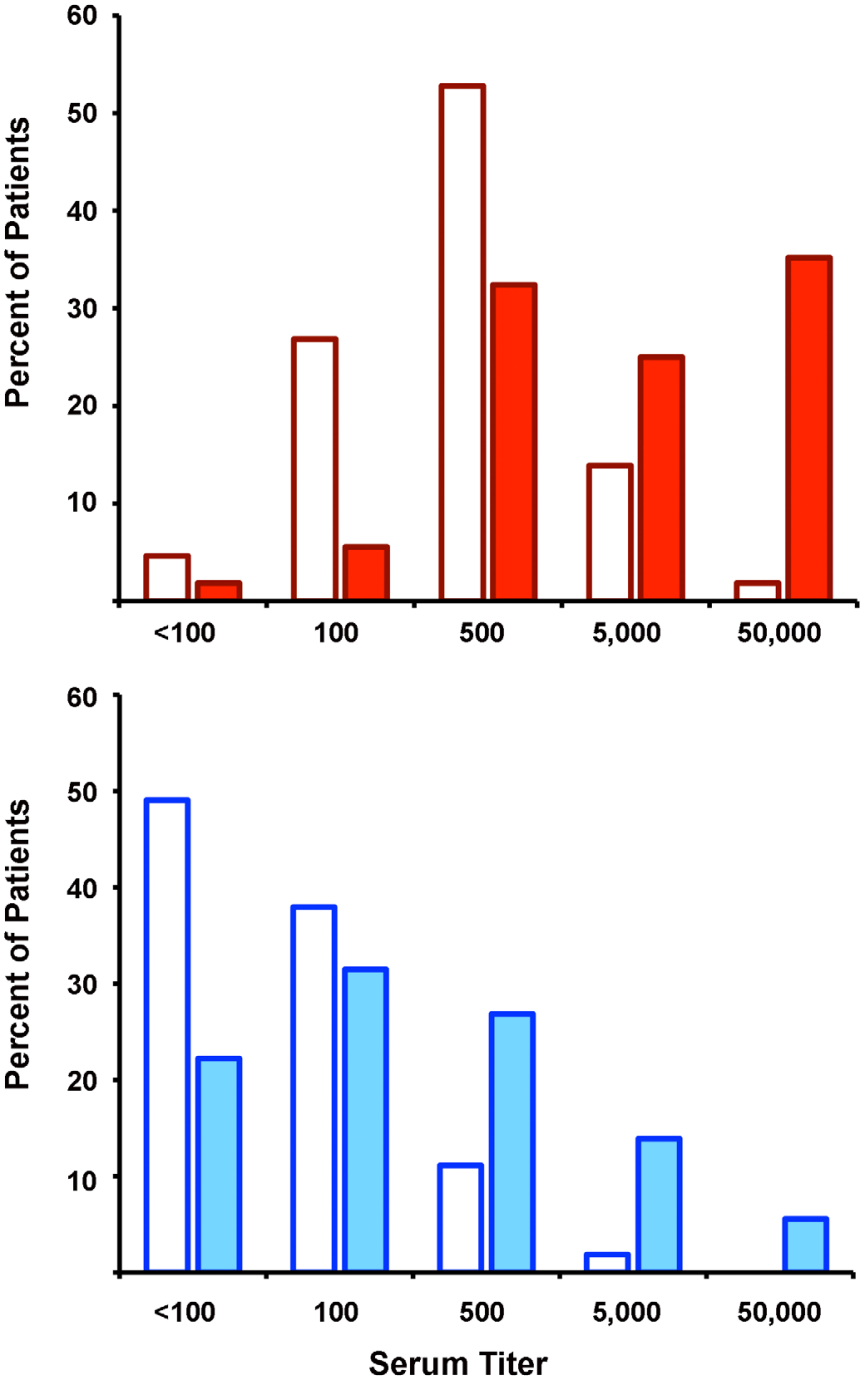
BKV-I and BKV-IV neutralizing titers in kidney transplant patients at study entry and exit. Sera from 108 kidney transplant recipients were titered for the presence of BKV-I (top panel) or BKV-IV (bottom panel) type-specific neutralizing antibodies. The percentage of patients at a particular titer cut-off at study entry (1 week after transplantation) are depicted as open bars, while the titers at study exit (1 year after transplantation) are shown as filled bars.

On average, the patients' BKV-I and BKV-IV neutralizing titers both increased substantially by one year after renal transplantation (Figure 4). In some instances, titer increases occurred even in patients who showed moderate neutralizing antibody titers at study entry (Figures 3 and S3). This result mirrors data that Bohl and colleagues obtained for this same set of sera using BKV-I VLP ELISAs [16]. It is unclear whether these titer increases reflect new cycles of infectious cell-to-cell spread or emergence of virion production from a latent reservoir, perhaps arising from the recipient's bladder epithelium or original kidneys.

## Discussion

In this report, we show that BKV-I and BKV-IV are distinct serotypes with respect to neutralizing antibody responses in human subjects. Until now, this concept would have been obscured by that fact that traditional VLP-based ELISAs, which have been used for prior investigations of BKV serology in human subjects, do not offer an accurate measurement of BKV serotype-specific neutralizing antibody responses.

Our longitudinal neutralization-based analysis of archived sera from 108 KTRs indicates that roughly half of the subjects were initially BKV-IV naïve at the time of transplantation. Roughly half of the initially seronegative subjects went on to show evidence of BKV-IV-specific seroconversion during the first year after transplantation. A simple model for this result would be that half of all adults are latently infected with BKV-IV and transplantation of a latently BKV-IV-infected kidney leads to opportunistic viral replication in BKV-IV-naïve KTRs. The concept that BKVN is a consequence of a *de novo* BKV infection arising from the engrafted kidney could help explain why the condition rarely affects recipients of other organ types, despite the use of similar immunosuppressive regimens [15].

An important implication of our findings is the possibility that induction of an effective BKV-IV-neutralizing antibody response prior to transplantation might protect some KTRs against outgrowth of BKV-IV harbored in the engrafted kidney. This might, in turn, prevent the development of pathological forms of BKV replication. The idea that BKV-IV may be disproportionately involved in BKVN is supported by studies showing that BKV viremia and viruria in KTRs is frequently attributable to BKV-IV [47]–[50].

Our results show that a single adjuvanted dose of BKV-IV VLPs can induce high titer BKV-IV-neutralizing antibody responses in experimentally vaccinated mice. This suggests that a BKV-IV VLP vaccine would likely be an effective way to elicit neutralizing antibody responses in prospective KTRs prior to the implementation of immunosuppressive therapy.

The potential value of pre-vaccination of KTRs could also extend to instances where donors and recipients are discordant for other BKV serotypes. In theory, elicitation of a broadly neutralizing antibody response against all BKVs might be accomplished using a vaccine containing VLPs based on multiple BKV serotypes. A precedent for this idea can be found in the vaccines Cervarix and Gardasil, which elicit neutralizing antibody responses against multiple HPV serotypes using mixtures of VLPs (reviewed in [51]). The clinical efficacy of current HPV vaccines correlates strongly with serological measurements using HPV neutralization assays [29]. This suggests that BKV neutralization assays, such as those reported here, could likewise serve as a useful proxy for the efficacy of candidate BKV vaccines.

Several previous studies have employed intravenous infusions of purified immunoglobulins (IVIG) in an attempt to suppress humoral responses to the engrafted organ and to possibly offer antibody-based suppression of BKV replication. The results of IVIG studies have been mixed [52]–[54]. In theory, this may have been due to differing levels and serotype specificities of BKV-neutralizing antibodies in various immunoglobulin preparations. A possible alternative approach to polyclonal IVIG would be to administer a cocktail of humanized monoclonal antibodies (mAbs) capable of neutralizing all BKV serotypes. Neutralization-based serology approaches should be useful for future determination of the total number of BKV serotypes and for the development of candidate anti-BKV therapeutic antibodies.

## Materials and Methods

### Ethics statement

Samples from 108 renal transplant subjects from the “Randomized Prospective Controlled Clinical and Pharmacoeconomic Study of Cyclosporine vs. Tacrolimus in Adult Renal Transplant Recipients” of the Washington University in St. Louis School of Medicine were used. For the original study, The Human Studies Committee of the Washington University School of Medicine approved the protocol and informed consent was obtained from all participants. For this study, the samples were assigned random identifier symbols prior to analysis at the National Cancer Institute (NCI).

All animal work was approved by the Animal Care and Use Committee of the NCI, according to the guidelines of the Association for Assessment and Accreditation of Laboratory Animal Care International. Procedures were carried out in accordance with the eighth edition of the National Research Council of the National Academies' Guide for the Care and Use of Laboratory Animals. All efforts were made to minimize animal suffering.

### Mice and immunization

Eight week old female BALB/cAnNCr mice were immunized once subcutaneously with 5 µg of BKV-I or BKV-IV virus-like particles (VLPs, see below) emulsified in complete Freund's adjuvant (CFA, Sigma). Sera were collected four weeks after immunization. The animals were kept under specific pathogen-free conditions in compliance with institutional guidelines at the National Cancer Institute (NCI).

### Sera

The clinical protocols used in this study have previously been described in detail [15], [17], [55]. Briefly, patients were given an immunosuppressive regimen, which was reduced if viremia was detected. Serum samples were collected at roughly 1, 4, 12, 26 and 52 weeks post-transplantation. None of the patients were observed to suffer from BKVN during the course of the collection period.

A previously-described set of sera from healthy subjects visiting U.S. plasma donation centers were purchased from Equitech Bio and Innovative Research [56].

### Reporter vectors and VLPs

BKV reporter vectors (pseudovirions) were generated as previously described [56]. BKV-I reporter vectors were produced using plasmid pCAG-BKV [35], which encodes the capsid proteins of BKV isolate KOM-5. KOM-5 is classified as a BKV type I subtype b-1 (Ib-1) genotype [57]. For BKV-IV particles, the sequence of BKV isolate A-66H (subtype IVc-2) [58] was used to design synthetic codon-modified VP1, VP2 and VP3 genes [36]. Capsid protein expression plasmids were co-transfected with a reporter plasmid encoding *Gaussia* luciferase (phGluc) into 293TT cells [34]. Forty eight hours after transfection, the cells were suspended at >100 million cells/ml in PBS and lysed by addition of 0.5% Triton X-100, and RNase A/T1 cocktail (Ambion). The lysate was incubated at 37°C overnight to allow capsid maturation, then clarified at 5,000× g. Reporter vector particles were purified out of the clarified supernatant by ultracentrifugation through a 27–33–39% iodixanol (Optiprep, Sigma) step gradient [59].

For generation of VLPs, 293TT cells were transfected with VP1/2/3 expression plasmids without any reporter plasmid. Two days after transfection, the cells were lysed with 0.5% Triton X-100 in DPBS supplemented with 25 mM ammonium sulfate, Benzonase (Sigma), Plasmid Safe (Epicentre) and 1.2 U/ml neuraminidase V (Sigma #N2876) [60], [61]. The lysates were incubated at 37°C overnight, then adjusted to 0.85 M NaCl, clarified as above and subjected to purification over Optiprep gradients.

Maps of the plasmids used in this work and detailed protocols for reporter vector and VLP production can be found at the following website: <http://home.ccr.cancer.gov/LCO/>. Plasmids are available through <http://www.AddGene.org/>.

### ELISAs

Immulon H2B plates (Thermo) were coated with 15 ng/well of VLPs in PBS overnight. PBS with 1% non-fat dry milk (blotto) was used to block the coated plates for 2 hours at room temperature, with orbital rotation. Sera from mice and healthy human subjects were serially diluted in blotto and incubated on blocked plates at room temperature for 1 hour, with orbital rotation. Washing was performed with PBS. Horseradish peroxidase conjugated goat anti-mouse IgG (BioRad) or donkey anti-human IgG (Jackson) secondary antibody diluted 1∶7500 in blotto was used to detect bound antibodies. The plates were incubated with ABTS substrate (Roche) and absorbance was read at 405 nm with a reference read at 490 nm. The effective concentration 10% (EC_10_) was calculated using Prism software (GraphPad) to fit a curve to the OD values for each serially diluted serum sample. The top of each dose-response curve was constrained based on the average of the calculated plateau maximum (Bmax) values for strongly reactive sera. The Bmax value was typically an OD of around 2.0, such that the EC_10_ value can be considered comparable to an OD cutoff value of 0.2.

### Neutralization assays

Neutralization assays were performed using previously reported methods [56]. Briefly, 293TT cells were seeded at a density of 3×10^4^ per well and allowed to attach for 3–5 hours. Sera from mice and human subjects were serially diluted, and sera from renal transplant patients were tested in separate assays at 4 different dilutions: 1∶100, 1∶500, 1∶5,000 and 1∶50,000. Dilutions were performed in cell culture medium (DMEM without phenol red supplemented with 25 mM HEPES, 10% heat-inactivated fetal bovine serum, 1% MEM non-essential amino acids, 1% Glutamax and 1% antibiotic-antimycotic, all from Invitrogen). Twenty-four µl of diluted sera were mixed with 96 µl of diluted reporter vector stock and placed at 4°C for 1 hour. One hundred µl of this mixture were added to the cells for 72 hours. Conditioned supernatants (25 µl) were harvested into white 96-well luminometry plates (Perkin Elmer). Fifty µl of *Gaussia* Luciferase Assay Kit substrate (NEB) were injected immediately prior to luminometry using a BMG Labtech Polarstar Optima luminometer. For sera from mice or from healthy human subjects, 50% neutralizing titers (EC_50_) were calculated based on dose-response curves with top and bottom values constrained to the average values of “no serum” and “no reporter vector” control wells, respectively. For transplant patients, the following criteria for seropositivity and seronegativity were adopted: sera were considered negative at entry if the 1∶100 dilution did not mediate at least a 95% reduction in *Gaussia* luciferase activity (measured in relative light units, RLUs) relative to the no serum control condition (i.e., >95% neutralization of the reporter vector). Seroconversion refers to subjects who scored seronegative at study entry but whose sera became >95% neutralizing at the 1∶500 dilution at any subsequent time point. A stricter definition of seroconversion, accounting for the possible low-level cross-type neutralization, added the stipulation that the 95% neutralizing titer for BKV-IV differed from the BKV-I neutralizing titer by less than 1,000-fold.

### Sequence analysis

VP1 protein sequences from BKV strains indicated in Figure S2 were downloaded from GenBank. ClustalW alignments were performed with MacVector software version 11.1.2 using a Gonnet series matrix. Structural modeling of BKV VP1 amino acid variations was performed by aligning the sequences of JCV or SV40 VP1 to BKV, followed by inspection of homologous positions of interest in the JCV or SV40 VP1 X-ray crystal structures (PDB ID accession codes 3NXD and 1SVA, respectively). Structure inspections were performed using Swiss PDB Viewer [62].

## Supporting Information

Figure S1 **BKV-I and BKV-IV ELISA vs. neutralizing titers in VLP-immunized mice and healthy adults.** In the top panels, the ELISA (x axis) or neutralizing titers (y axis) for six BKV-I (red circles) or BKV-IV (blue squares) VLP immunized mice are shown. Neutralizing titers against the BKV-I pseudovirus are shown in the top left panel, and anti-BKV-IV titers are in the top right. The bottom panels show similar titer comparisons for sera from healthy adults.
(TIF)

Figure S2 **Alignment of BKV VP1 proteins.**
(PDF)

Figure S3 **BKV-I and BKV-IV neutralization patterns in kidney transplant recipients.** Sera from kidney transplant recipients were titered for the presence of BKV-I (red circles) or BKV-IV (blue squares) neutralizing antibodies. The neutralizing titer categories shown on the y axis are defined as 1) <95% neutralization at a serum dilution of 1∶100, 2) ≥95% neutralization at 1∶100, 3) ≥95% neutralization at 1∶500, 4) ≥95% neutralizing at 1∶5,000, and 5) ≥95% neutralizing at 1∶50,000. Sera were collected at 5 different time points (x axis) spanning roughly 1, 4, 12, 26, and 52 weeks post-transplantation (designated letters A-E, respectively). The patterns of all 108 patients in the study are shown. In each panel, the notations in the bottom right corner represent the BKV genotype (I, II, or IV) detected in the patient's urine (superscript u) or blood (superscript b) at or after the observed onset of viruria. The numbers at the top of each graph denote quantitation of BKV viruria (log_10_ BKV DNA copies per ml) at each time point. Dashes indicate that BKV DNA was not detected in the urine. The symbol “nr” indicates no results for the time point. The symbol “utq” indicates that the BKV viruria signal was too low for accurate quantitation. Asterisks mark time points at which BKV viremia was quantitated. The symbol JC+ indicates that JC virus DNA was detected.
(PDF)
