# Supplementary figures and images for: Neutralization Serotyping of BK Polyomavirus Infection in Kidney Transplant Recipients
Source: PLoS Pathog. 2012 Apr 12;8(4):e1002650. doi: 10.1371/journal.ppat.1002650 (PMC3325208; doi:10.1371/journal.ppat.1002650)

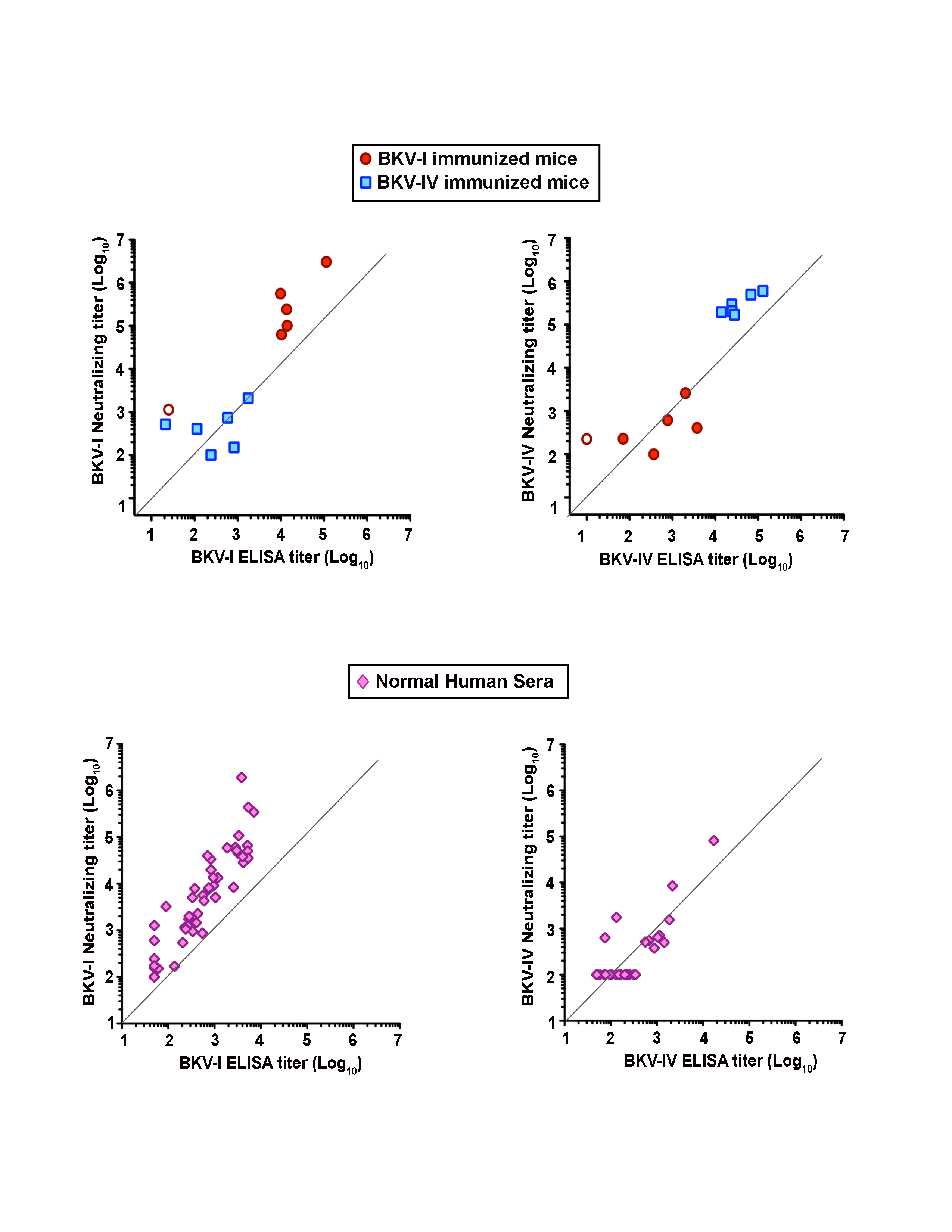

Supplement: Figure S1 — BKV-I and BKV-IV ELISA vs. neutralizing titers in VLP-immunized mice and healthy adults. In the top panels, the ELISA (x axis) or neutralizing titers (y axis) for six BKV-I (red circles) or BKV-IV (blue squares) VLP immunized mice are shown. Neutralizing titers against the BKV-I pseudovirus are shown in the top left panel, and anti-BKV-IV titers are in the top right. The bottom panels show similar titer comparisons for sera from healthy adults. (TIF) [file ppat.1002650.s001.tif]

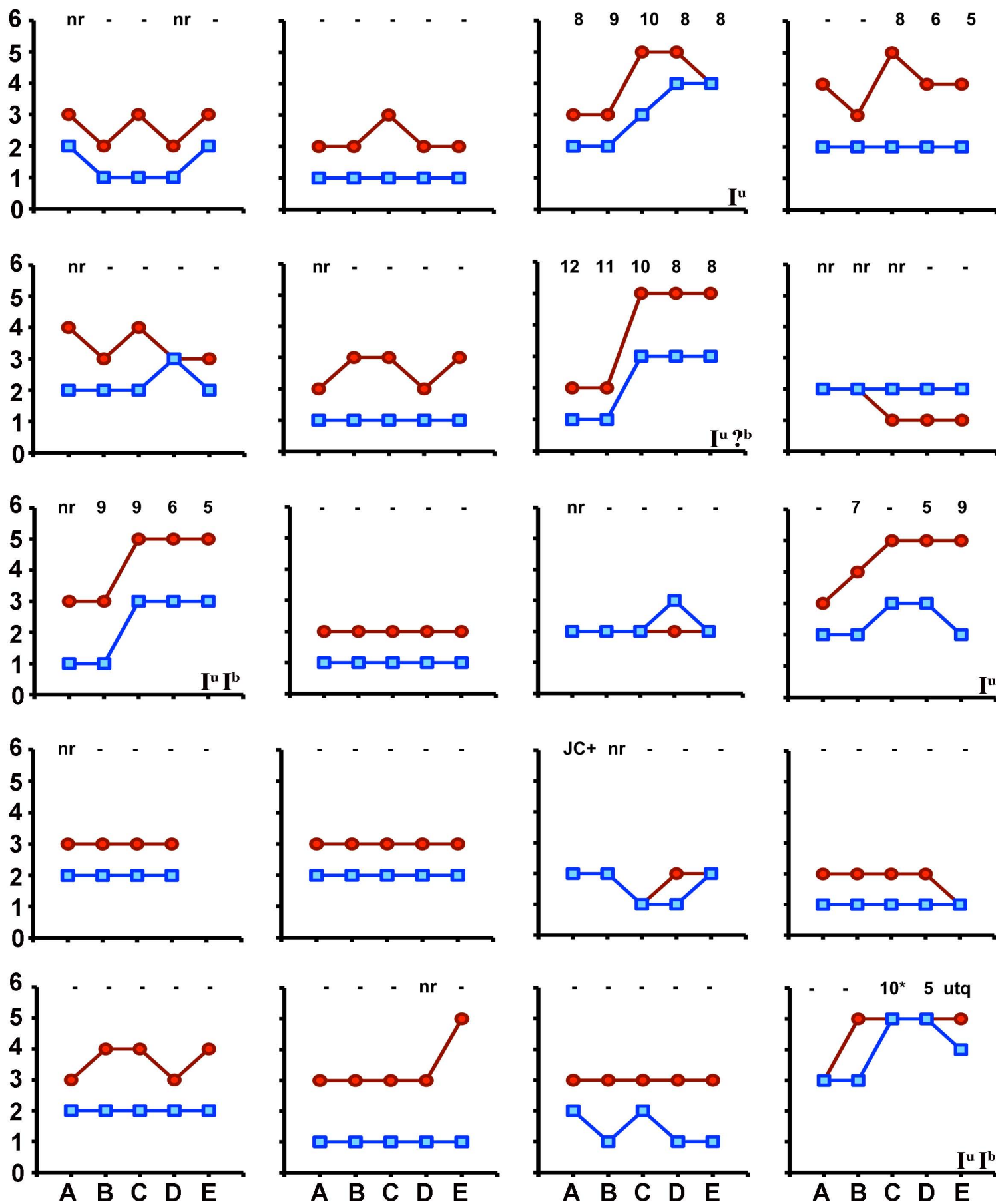

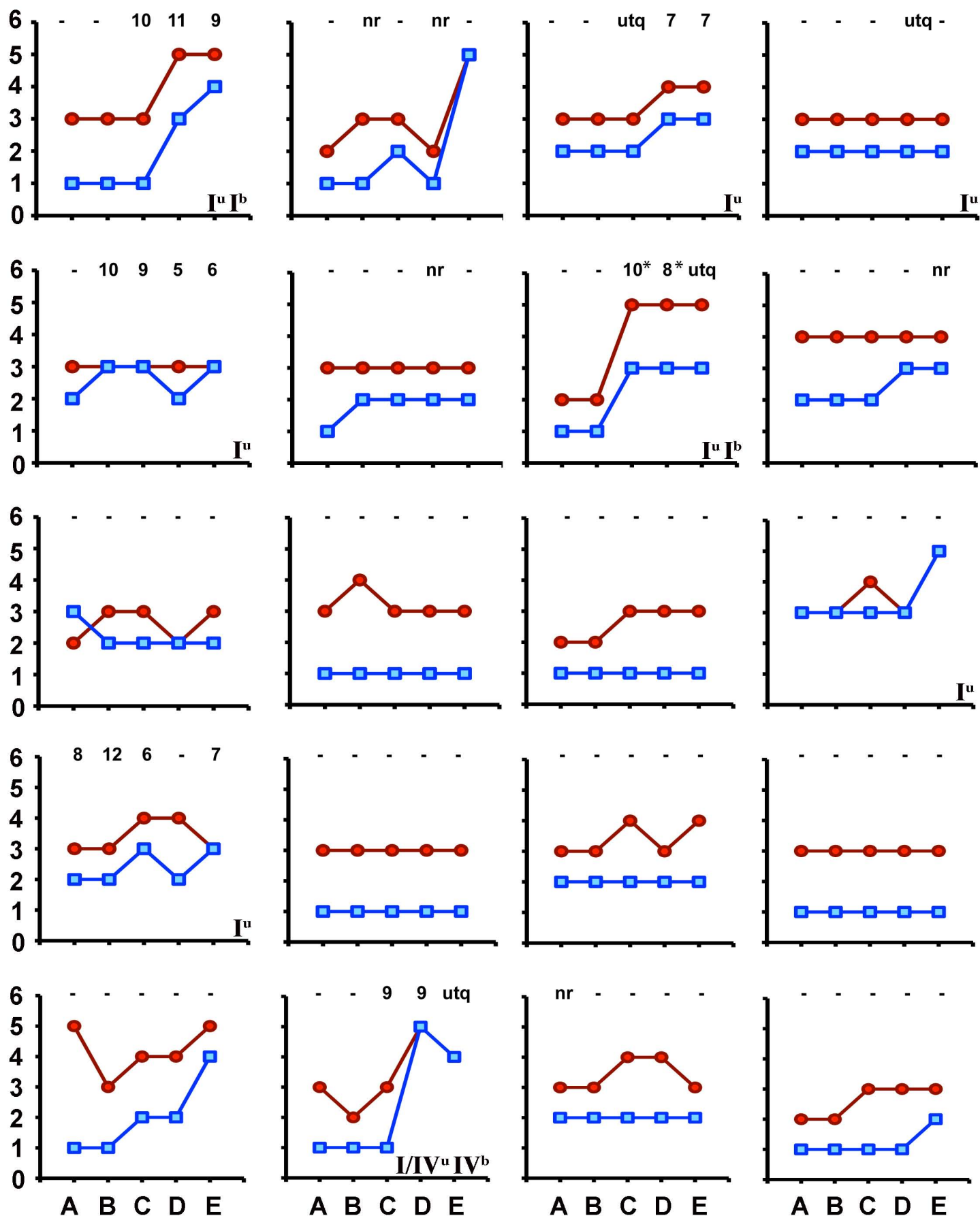

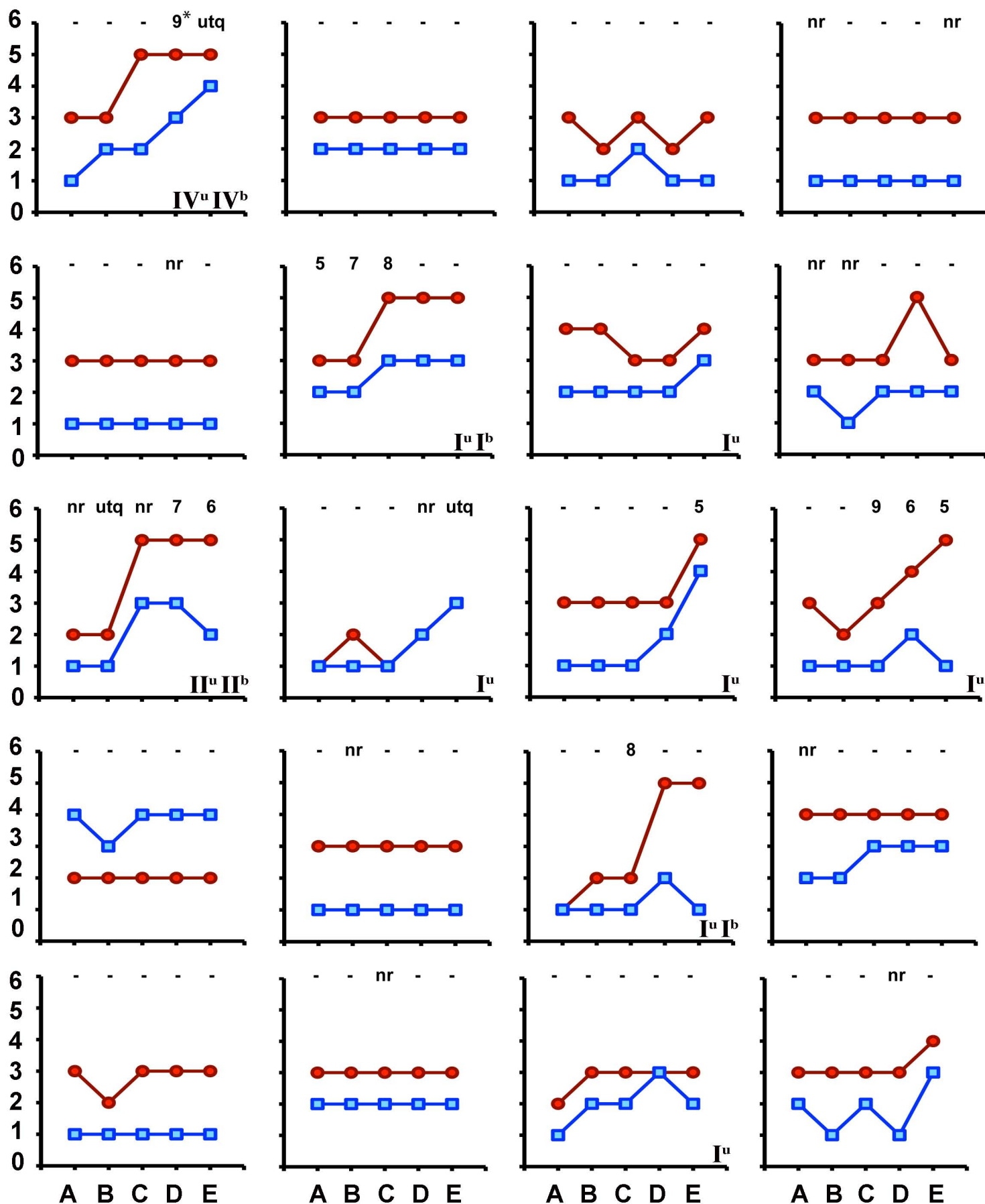

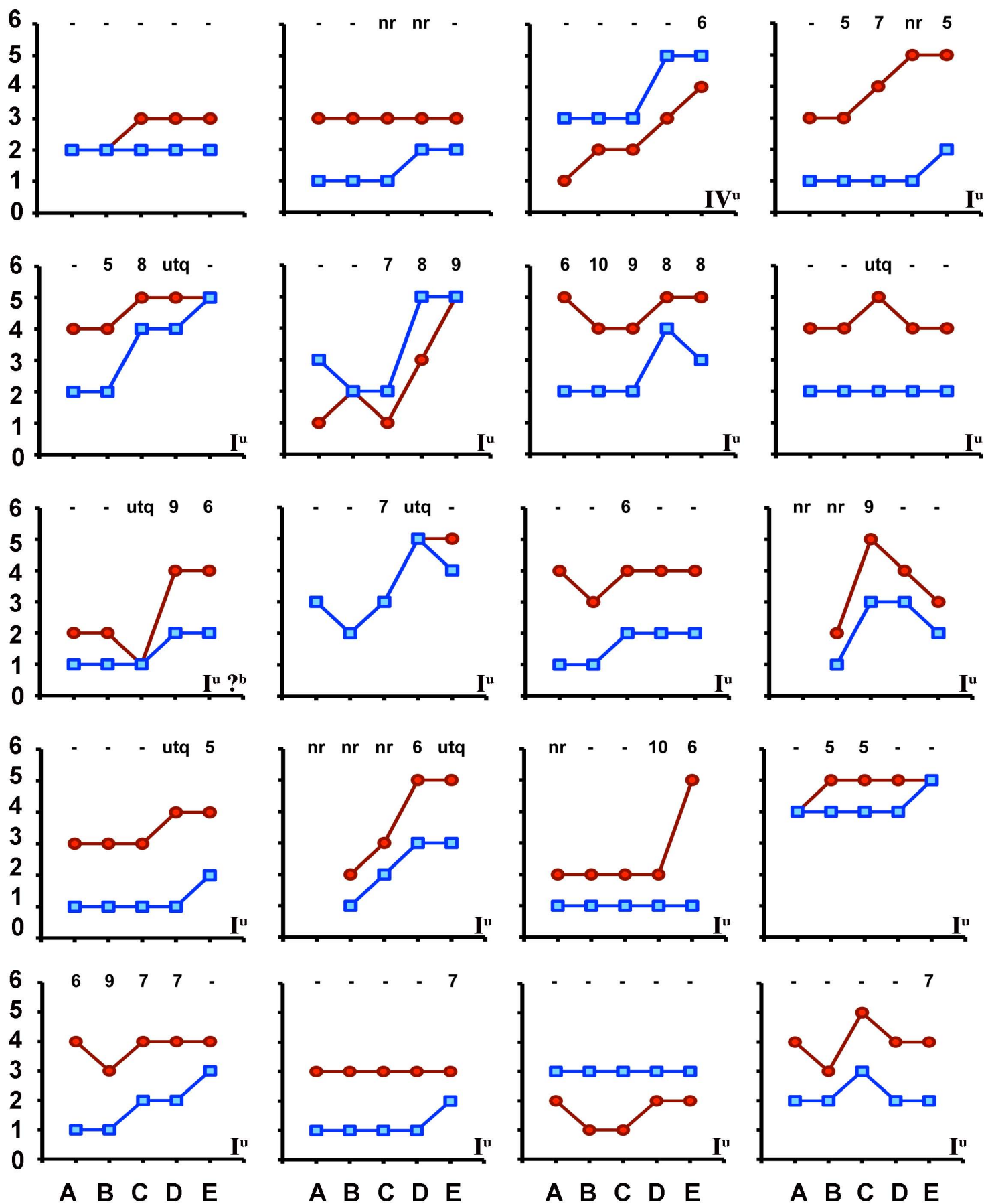

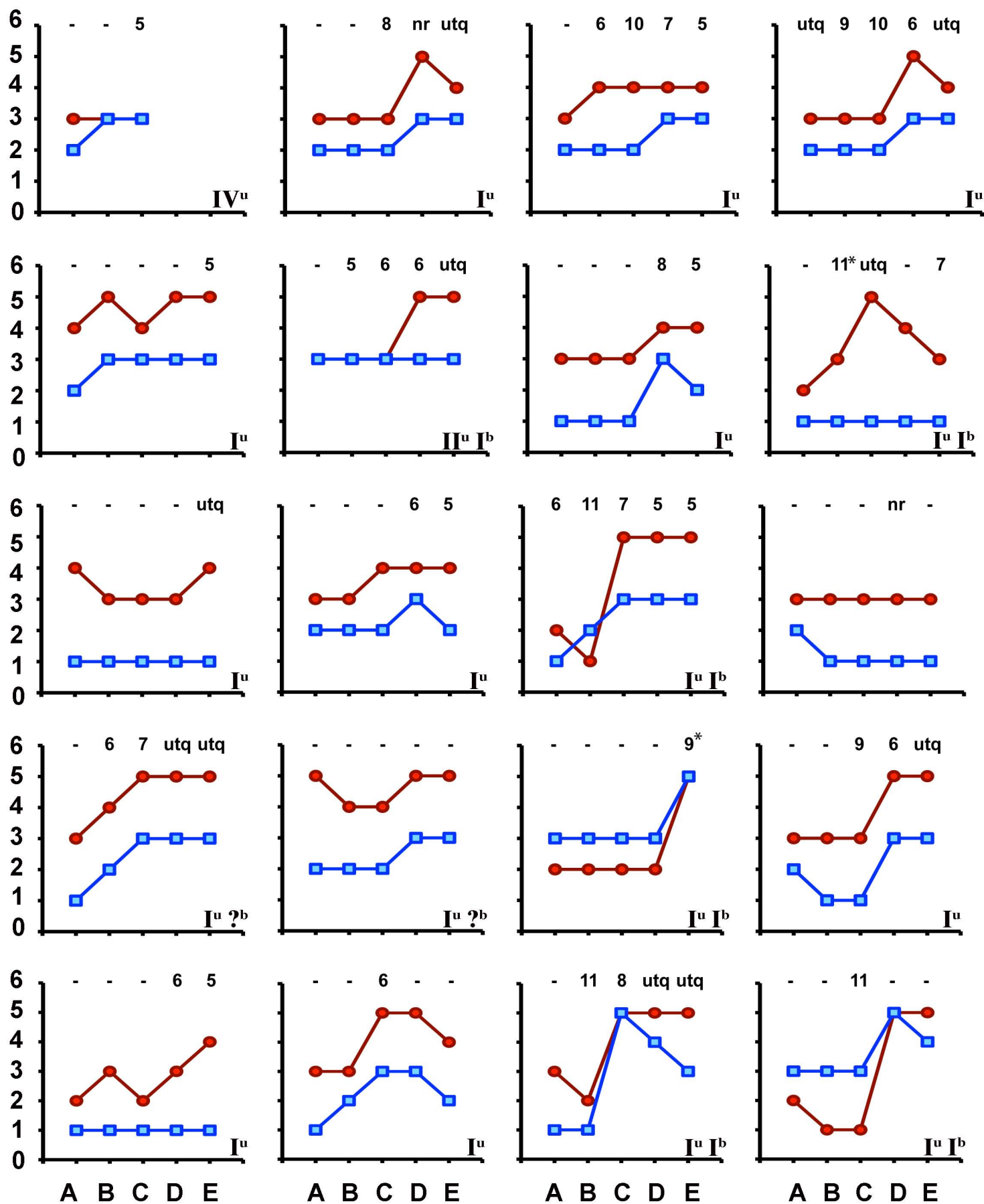

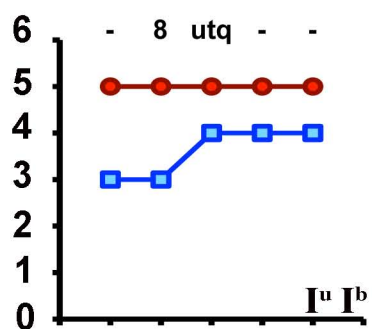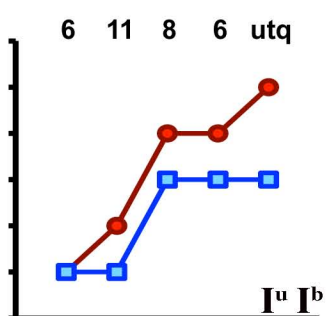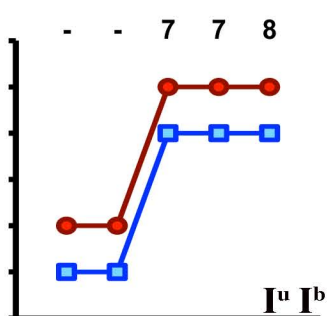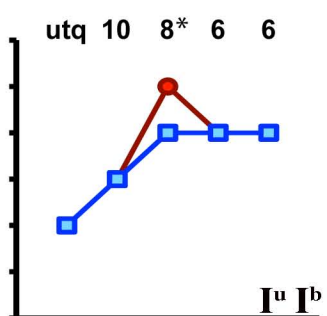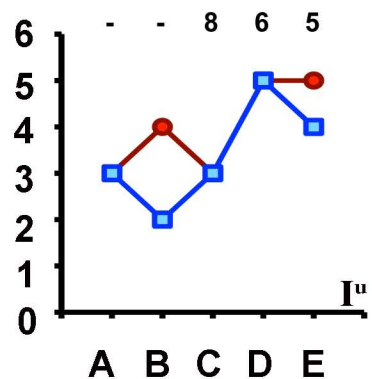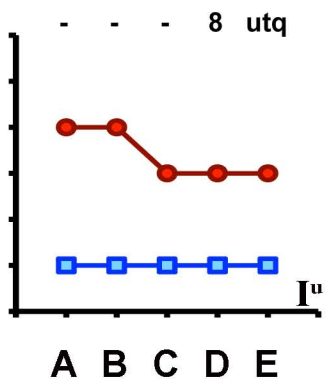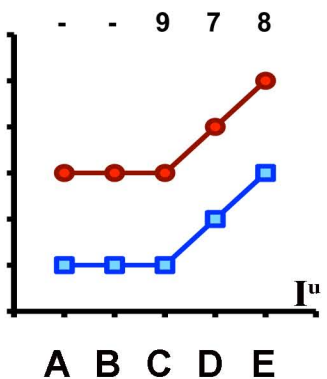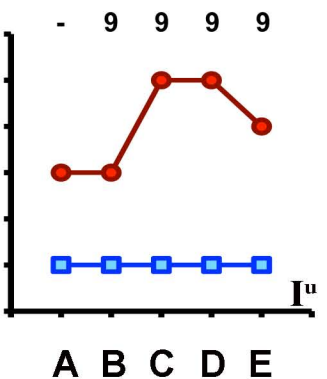

Supplement: Figure S3 — BKV-I and BKV-IV neutralization patterns in kidney transplant recipients. Sera from kidney transplant recipients were titered for the presence of BKV-I (red circles) or BKV-IV (blue squares) neutralizing antibodies. The neutralizing titer categories shown on the y axis are defined as 1) <95% neutralization at a serum dilution of 1∶100, 2) ≥95% neutralization at 1∶100, 3) ≥95% neutralization at 1∶500, 4) ≥95% neutralizing at 1∶5,000, and 5) ≥95% neutralizing at 1∶50,000. Sera were collected at 5 different time points (x axis) spanning roughly 1, 4, 12, 26, and 52 weeks post-transplantation (designated letters A-E, respectively). The patterns of all 108 patients in the study are shown. In each panel, the notations in the bottom right corner represent the BKV genotype (I, II, or IV) detected in the patient's urine (superscript u) or blood (superscript b) at or after the observed onset of viruria. The numbers at the top of each graph denote quantitation of BKV viruria (log10 BKV DNA copies per ml) at each time point. Dashes indicate that BKV DNA was not detected in the urine. The symbol “nr” indicates no results for the time point. The symbol “utq” indicates that the BKV viruria signal was too low for accurate quantitation. Asterisks mark time points at which BKV viremia was quantitated. The symbol JC+ indicates that JC virus DNA was detected. (PDF) [file ppat.1002650.s003.pdf]
