# Supplementary material for: Neutralization Serotyping of BK Polyomavirus Infection in Kidney Transplant Recipients
Source: PLoS Pathog. 2012 Apr 12;8(4):e1002650. doi: 10.1371/journal.ppat.1002650 (PMC3325208; doi:10.1371/journal.ppat.1002650)

Formatted Alignments

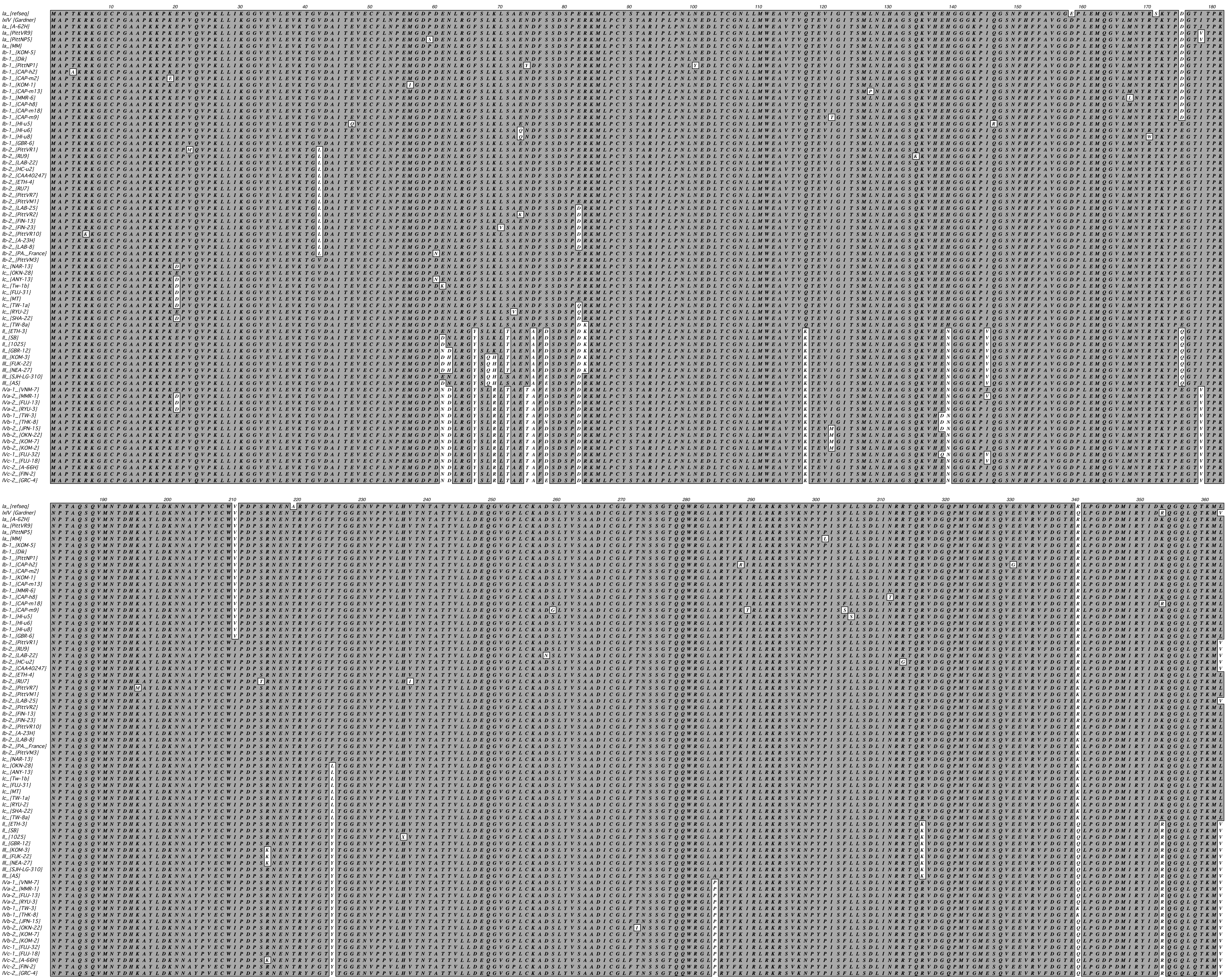

Supplement: Figure S2 — Alignment of BKV VP1 proteins. (PDF) [file ppat.1002650.s002.pdf]
